# Supplementary material for: Comparative genomics provides new insights into the diversity, physiology, and sexuality of the only industrially exploited tremellomycete: Phaffia rhodozyma
Source: BMC Genomics. 2016 Nov 9;17:901. doi: 10.1186/s12864-016-3244-7 (PMC5103461; doi:10.1186/s12864-016-3244-7)
Supplement: Additional file 6: — List of orphan genes with links to PFAM (related to Additional file 1: Table S1). (ZIP 1428 kb) [file 12864_2016_3244_MOESM6_ESM.zip › PFAM_UNIQUE/G01111_P.html]

#                                                                            --- full sequence --- -------------- this domain -------------   hmm coord   ali coord   env coord
  
# target name        accession   tlen query name           accession   qlen   E-value  score  bias   #  of  c-Evalue  i-Evalue  score  bias  from    to  from    to  from    to  acc description of target
  
#------------------- ---------- ----- -------------------- ---------- ----- --------- ------ ----- --- --- --------- --------- ------ ----- ----- ----- ----- ----- ----- ----- ---- ---------------------
  
G01111\_P             -            149 ARGLU                PF15346.1    149       6.8    5.1   7.5   1   1     0.026       9.6    4.6   7.5    68   124    86   140    70   146 0.51 - Arginine and glutamate-rich 1  
G01111\_P             -            149 Cast                 PF10174.4    775    0.0091   12.8   2.8   1   1   4.7e-05      0.01   12.6   2.8   449   510    81   142    61   147 0.88 - RIM-binding protein of the cytomatrix active zone  
G01111\_P             -            149 Cortex-I\_coil        PF09304.5    107     0.091   11.5   1.0   1   1    0.0003      0.17   10.7   1.0    74   105    94   125    71   127 0.85 - Cortexillin I, coiled coil  
G01111\_P             -            149 CR6\_interact         PF10147.4    217       1.5    6.7   6.3   1   1    0.0039       2.1    6.3   6.3   106   163    82   139    73   145 0.90 - Growth arrest and DNA-damage-inducible proteins-interacting protein 1  
G01111\_P             -            149 DUF4659              PF15558.1    376       0.8    6.9   8.1   1   1    0.0016         1    6.6   8.1    92   150    79   140    71   145 0.75 - Domain of unknown function (DUF4659)  
G01111\_P             -            149 ERM                  PF00769.14   246         2    6.5   7.2   1   1     0.017       2.5    6.2   7.2    70   129    85   144    72   147 0.84 - Ezrin/radixin/moesin family  
G01111\_P             -            149 MAP7                 PF05672.6    171       0.5    8.4   6.4   1   1    0.0025      0.63    8.1   6.4    34    94    81   143    59   146 0.67 - MAP7 (E-MAP-115) family  
G01111\_P             -            149 Transposase\_22       PF02994.9    370     0.021   12.0   1.6   1   1   3.5e-05     0.026   11.7   1.6    61   119    81   139    68   145 0.87 - L1 transposable element  
G01111\_P             -            149 Troponin             PF00992.15   132       3.7    6.3   6.8   1   1    0.0064       5.5    5.7   6.8    18    58    89   130    84   145 0.77 - Troponin
